# Immunological Profile in Atypical Kawasaki Disease: A Case Report Highlighting the Diagnostic Utility of Cytokine Analysis by qRT-PCR

**DOI:** 10.3390/pediatric17060128

**Published:** 2025-12-01

**Authors:** Margarita L. Martinez-Fierro, Idalia Garza-Veloz, Felipe D. Marrufo-Garcia, Manuel Gonzalez-Plascencia, Rocio C. Calderon-Zamora, Claudia Sifuentes-Franco, Monica Rodriguez-Borroel

**Affiliations:** 1 Molecular Medicine Laboratory, Academic Unit of Human Medicine and Health Sciences, Universidad Autonoma de Zacatecas, Carretera Zacatecas-Guadalajara Km.6. Ejido la Escondida, Zacatecas 98160, Mexico; idaliagv@uaz.edu.mx (I.G.-V.); garciafelipe894@gmail.com (F.D.M.-G.); manuelgonzalezcharro@gmail.com (M.G.-P.); 2 Hospital General de Zona No.1, Mexican Social Security Institute for Welfare (IMSS-BIENESTAR), Zacatecas 98040, Mexico; rc.calderon@live.com (R.C.C.-Z.); claussf03@hotmail.com (C.S.-F.)

**Keywords:** Kawasaki disease, cytokine profile, vasculitis, viral infection

## Abstract

**Background:** Kawasaki Disease (KD) is an acute vasculitis affecting children under five years of age, with atypical presentations posing diagnostic challenges and a higher risk of coronary complications when untreated. **Methods:** We report on a 2-year-old girl with persistent fever, limb edema, erythema, and non-purulent conjunctivitis, without cervical lymphadenopathy or the typical rash. Inflammatory markers were assessed, and a cytokine expression profile was obtained using qRT-PCR. **Results:** Laboratory analysis showed elevated C-reactive protein (11.1 mg/dL), high fibrinogen (468 mg/dL), borderline D-dimer (484 ng/mL), and a normal platelet count. The cytokine profile revealed marked upregulation of IFN-α, IFN-β, IFN-γ, IL-1α, IL-1β, IL-5, IL-8, and IL-12, with downregulation of IL-2 and IL-4, as well as low TNF-α levels. These findings, although not pathognomonic, were consistent with an inflammatory profile compatible with atypical KD, in which a preceding viral infection may have played a role, although causality cannot be established. **Conclusions:** This case highlights the diagnostic utility of cytokine profiling in suspected atypical KD, particularly when clinical criteria are incomplete. The integration of immunological data may aid in earlier recognition and therapeutic intervention, thereby helping to prevent cardiovascular sequelae. Cytokine analysis may serve as a promising adjunct for atypical KD diagnosis, although confirmation in larger cohorts is needed.

## 1. Introduction

Kawasaki disease (KD) is an acute systemic vasculitis that predominantly affects children under five years of age, although the disease can occur even in adolescence. It is initially manifested by high fever, mucocutaneous inflammation, and cervical lymphadenopathy and represents the leading cause of acquired cardiovascular disease in childhood in developed countries; without treatment, patients develop coronary artery dilatation or coronary artery (CA) aneurysms [1,2,3].

It was first described in Japan in 1967 by Tomisaku Kawasaki in a paper wherein he described 50 patients with systemic features and prolonged fever [4]. The disease has since been recognized globally, with a particularly high incidence in East Asia [2,4]. Classic KD is characterized by persistent fever lasting at least five days (greater than 38 °C), accompanied by at least four of the following clinical signs: bilateral non-purulent conjunctivitis, changes in the oral mucosa (fissured lips, strawberry tongue, or a red pharynx), polymorphous rash, cervical lymphadenopathy, and changes in the extremities (redness of the palms and soles, acute-phase edema of the hands and feet, and peeling of the skin around the nails after the acute phase) [1,5]. The definition of atypical KD should be reserved for patients who have clinical manifestations such as renal impairment, unilateral peripheral facial nerve palsy, testicular swelling, pulmonary nodules and/or infiltrates, pleural effusions, diarrhea, vomiting, abdominal pain, acute surgical abdomen, and hemophagocytic syndrome that generally are not seen in KD [6]. However, between 15% and 36.2% of pediatric cases present as incomplete or atypical forms, which complicates timely recognition [7]. These forms, which do not meet all classical criteria, are more common in infants under 12 months and in children older than 5 years [8]. Since delays in diagnosis and treatment increase the risk of coronary aneurysms, it is essential to have auxiliary diagnostic tools to guide clinical decision-making. In this context, analysis of the immunological profile using molecular biology techniques such as quantitative reverse transcription polymerase chain reaction (qRT-PCR) represents a promising tool to characterize the patient’s inflammatory response [9,10]. The detection of specific cytokine patterns, such as overexpression of type I interferons (IFN-α, IFN-β), proinflammatory interleukins (IL-1α, IL-1β, IL-8, IL-12), and a Th1 bias (IFN-γ), can provide valuable insights into the underlying pathophysiology and help differentiate atypical KD from other entities such as lupus, eosinophilic vasculitis, or multisystem inflammatory syndromes associated with viral infections [11,12,13,14,15,16,17,18]. This case report aims to describe the case of an infant patient with suspected atypical KD, in whom the immunological profile obtained through qRT-PCR provided relevant evidence for diagnostic consideration. Furthermore, it proposes a clinical-diagnostic approach based on cytokine expression patterns and discusses its potential utility for improving early detection of incomplete forms in pediatric practice.

## 2. Case Description

**Figure 1:**
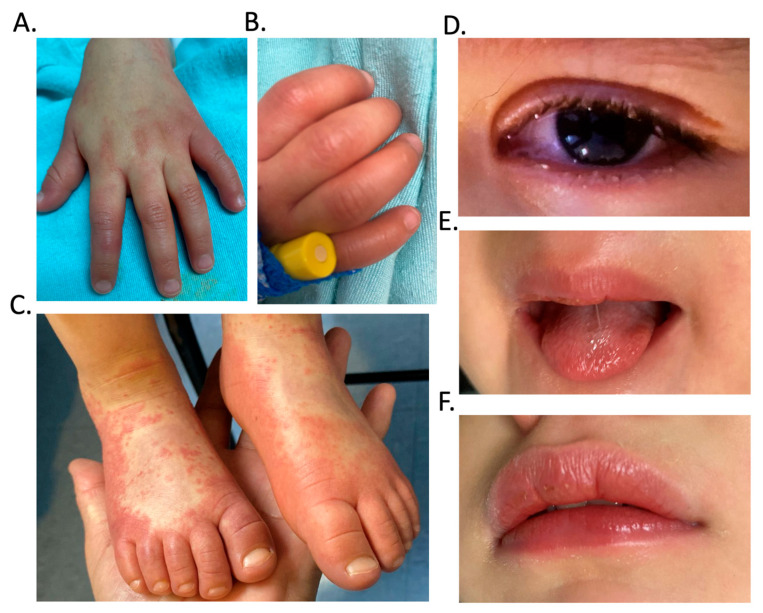
Clinical findings in atypical Kawasaki disease. Images (**A**,**B**) show maculopapular erythema and edema on the upper extremity. Image (**C**) shows maculopapular erythema and edema on the lower extremity. Image (**D**) shows bilateral non-purulent conjunctival injection with hyperemia. Image (**E**) shows a furred tongue with a strawberry-like appearance. Image (**F**) shows cracked and edematous lips.

A previously healthy two-year-old girl was brought to the emergency department with a two-day history of persistent fever reaching 39 °C accompanied by maculopapular erythema that began on the left lower extremity (foot) and right upper extremity (hand) (Figure 1A–C) and subsequently spread to the chest, abdomen, and buttocks; indurated edema in both pelvic and thoracic extremities with clear signs of redness, heat, and pain on palpation (Figure 1B); bilateral non-purulent conjunctival injection (Figure 1D); and vomiting. She was taken to the pediatric emergency department, where inflammation of the oral mucosa and cracked lips were also observed (Figure 1E,F), as well as clear rhinorrhea. No lymphadenopathy was observed at that time. Figure 1 summarizes the clinical findings in the patient. On day one of hospitalization, defined as the day of hospital admission (corresponding to day 2 of fever), initial blood and urine samples were obtained. The complete blood count values are detailed in Table 1. At the time of sampling, the patient presented with normocytic normochromic anemia (Hb 10.9 g/dL), neutrophilia (9.2 × 10^3^/µL), and lymphopenia (1.7 × 10^3^/µL), along with elevated acute-phase reactants, including C-reactive protein (CRP; 11.10 mg/dL) and erythrocyte sedimentation rate (ESR; 45 mm/h), findings that are characteristic of an acute inflammatory response. The coagulation profile demonstrated prolonged partial thromboplastin time (66.2 s) and increased fibrinogen levels (468 mg/dL). Liver function tests were within normal limits, except for a mild elevation in alkaline phosphatase (343.5 U/L) and alanine aminotransferase (35 U/L) (Table 1). Urine culture yielded no bacterial growth, and the findings of the general urinalysis are detailed in Table 2. Chest radiography revealed an interstitial infiltrate predominantly in the right parahilar region, without evidence of cardiomegaly.

Given the history of fever, erythema, conjunctival injection, and oral changes, Kawasaki disease (KD) was suspected. However, because not all diagnostic criteria were fulfilled, a diagnosis of atypical KD was established in accordance with the American Heart Association (AHA) guidelines. Treatment was initiated on the day of hospital admission with intravenous immunoglobulin (IVIG, 2 g/kg) and aspirin (ASA, 50 mg/kg/day). The patient was referred to the pediatric department for further treatment. In view of the suspected acute-phase systemic vasculitis without complete KD criteria, an immunological profile was assessed by qRT-PCR on the day of hospital admission (day 2 of fever), prior to IVIG administration. For that, peripheral blood samples were collected, and peripheral mononuclear cells (PBMCs) were isolated by density gradient centrifugation using Histopaque^®^-1077 (Sigma-Aldrich, St. Louis, MO, USA).

**Figure 2:**
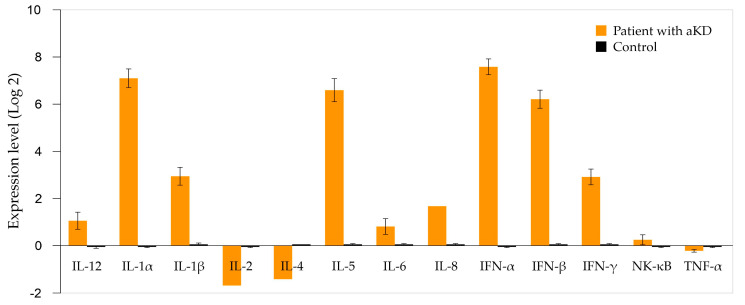
Peripheral blood mononuclear cell cytokine expression profile in atypical Kawasaki disease. The figure shows the expression levels of a 13-cytokine panel in a patient with atypical Kawasaki disease. Gene expression was quantified by quantitative real-time polymerase chain reaction (qRT-PCR), using GAPDH as an endogenous control and RNA extracted from peripheral blood mononuclear cells (PBMCs) of three healthy controls (negative for SARS-CoV-2 by qRT-PCR) as a calibrator. aKD: atypical Kawasaki disease.

Total RNA was extracted using the commercial PureLink RNA Mini Kit (Thermo Fisher Scientific, Waltham, MA, USA), followed by reverse transcription using the High-Capacity cDNA Reverse Transcription Kit (Applied Biosystems, Foster City, CA, USA). Relative gene expression of cytokines was quantified by real-time polymerase chain reaction (qRT-PCR) using SYBR Green Master Mix on a 7500 Fast Dx Real-Time PCR system (Applied Biosystems). Negative controls and calibrators were included, and expression was normalized against the reference gene GAPDH using the 2^−ΔΔCq^ method. Expression results were reported as log 2 fold change, using the mean values from three healthy donors as calibrator controls (confirmed SARS-CoV-2-negative by qRT-PCR). Thresholds were defined as follows: log 2 ≥ 1.0 indicates overexpression; log 2 ≤ −1.0 indicates underexpression; values between –1 and 1 reflect no significant differential expression. All reactions were run in triplicate, and melting curve analysis was used to ensure specificity. Figure 2 shows the results.

Upregulation was observed in IFN-α (7.71 ± 0.35), IFN-β (6.30 ± 0.35), IL-1α (7.19 ± 0.43), IL-1β (2.96 ± 0.40), IL-5 (6.68 ± 0.52), IL-8 (1.64 ± 0.001), IL-12 (1.03 ± 0.37), and IFN-γ (2.94 ± 0.32). In contrast, IL-2 (−1.69 ± 0.001), IL-4 (−1.41 ± 0.001), and TNF-α (−0.22 ± 0.04) were downregulated, while NF-κB (0.21 ± 0.22) and IL-6 (0.77 ± 0.32) showed moderately elevated expression. These findings were integrated with the clinical context, supporting the suspicion of atypical KD in the early acute phase. In parallel, protein quantification by ELISA for three selected cytokines (IL-6, IL-5, and TNF-α; Table 1) confirmed the transcriptional directionality: IL-6 at 2.19 pg/mL (ref: 0–7 pg/mL), IL-5 at 8.24 pg/mL (ref: 2–10 pg/mL), and TNF-α at 0.88 pg/mL (ref: 0.5–1.4 pg/mL).

Because cytokine expression data were derived from a single PBMC sample of the patient collected at a single time point, no statistical inference was applied, and results are descriptive. However, as part of the immunological analysis, a comparative matrix was constructed to contrast the patient’s cytokine expression profile with canonical patterns reported in three major diagnostic categories: classic KD, atypical/incomplete KD, and post-viral immunologic syndromes such as MIS-C. Each cytokine was categorized based on literature-supported behavior (↑ upregulation, ↓ downregulation, ↔ baseline levels), enabling a side-by-side comparison with the quantitative data obtained from qRT-PCR. Directional arrows in Table 3 illustrate the expected versus observed transcriptional behavior and facilitate rapid differential alignment. For instance, during the acute phase of classic KD, TNF-α and IL-6 are commonly upregulated and have been implicated in coronary complications. In contrast, our patient showed downregulation of TNF-α (−0.22) and moderate IL-6 (0.77), but a pronounced type I interferon signature (IFN-α: 7.71; IFN-β: 6.30) along with strong IL-1α (7.19) and IL-1β (2.96) expression. Such a skewed Th1/interferon-driven profile diverges from post-viral inflammatory syndromes like MIS-C, which exhibit prominent TNF-α and IL-10 elevations.

**Figure 3:**
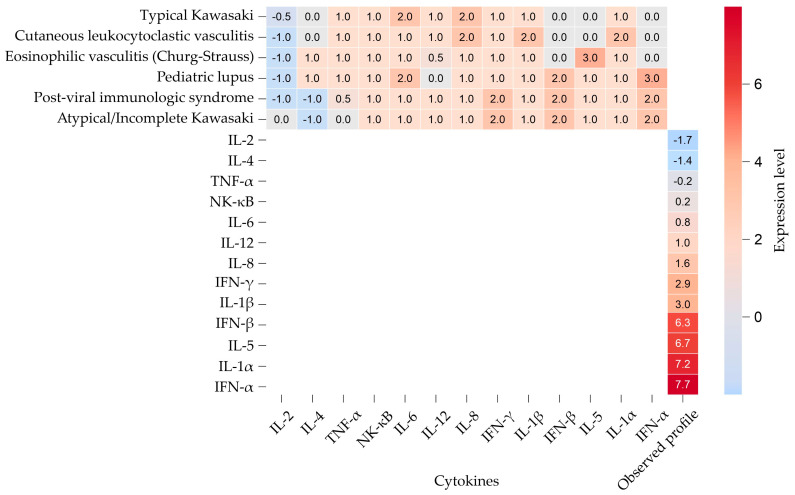
Heatmap of cytokine expression in pediatric vasculitis and differential diagnoses. This heatmap displays the relative expression levels of key cytokines in the reported patient (bottom row: “Observed profile”) compared with typical immunological patterns seen in major pediatric vasculitis-related conditions, including typical and atypical Kawasaki disease (KD), post-viral immunologic syndromes, pediatric lupus, cutaneous leukocytoclastic vasculitis, and eosinophilic vasculitis (Churg–Strauss). Red shades indicate upregulation, blue shades indicate downregulation, and white denotes near-normal levels. The observed profile demonstrates a mixed interferon/Th1-dominant signature with high levels of IFN-α, IFN-β, IL-1α/β, IL-8, IL-12, and IFN-γ, and downregulation of IL-2, IL-4, and TNF-α, supporting the diagnosis of KD with a potential post-viral trigger. In this comparative analysis, incomplete and atypical KD are jointly labeled as a single category, “atypical/incomplete KD”, reflecting the lack of clear immunological delineation between these forms.

Considering the data in Table 3, a heatmap was constructed to visually compare the cytokine expression profile of the patient with typical patterns reported in major pediatric vasculitis entities (Figure 3). For this purpose, we assigned semiquantitative ordinal values to reported cytokine expression patterns: “↑” was coded as +1, “↑↑” as +2, and “↑↑↑” as +3; “↓” was coded as −1 and “↓↓” as −2; “↔” (no change or normal) was coded as 0; and intermediate or variable levels such as “↔/↑” were coded as +0.5. These codes allowed a direct numeric comparison of qualitative trends with the patient’s actual qRT-PCR fold-change values, which were also included as a reference row in the heatmap. In the heatmap, color intensity corresponds to the direction and magnitude of expression: shades of red represent upregulation, shades of blue indicate downregulation, and white corresponds to near-normal levels. The profile of the patient demonstrated strong overexpression of type I interferons (IFN-α, IFN-β), IL-1α, IL-1β, IL-5, IL-8, IL-12, and IFN-γ, with downregulation of IL-2, IL-4, and TNF-α. When matched against the reference profiles, this pattern closely aligned with atypical KD but also partially overlapped with features of post-viral inflammatory syndromes. The visual comparison highlights the potential diagnostic utility of cytokine-based heatmaps as tools to support differential diagnosis in cases lacking full clinical criteria.

On the second day of hospitalization, a nasopharyngeal sample was taken for a rapid COVID-19 test, which tested negative; therefore, the viral panel was broadened using the multiplex PCR system to include types 2, 3, and 4 of human parainfluenza virus, adenovirus, coronavirus 229E, and rhinovirus using the FilmArray^®^ Respiratory Panel (BioFire Diagnostics, bioMérieux, Salt Lake City, UT, USA). Results were expected within two days, yielding a positive rhinovirus result on day 4 of hospitalization (Table 4). In addition, the patient was observed to have cracked lips and a coated tongue with strawberry-like margins. Due to the risk of cardiac complications and as part of a multidisciplinary approach, the patient was also evaluated by the pediatric cardiology service, which performed an initial two-dimensional transthoracic echocardiogram that showed mild pericardial effusion without hemodynamic repercussions; preserved biventricular function; main coronary arteries without alterations (right coronary artery with a Z-score of 0.18, left main coronary artery with a Z-score of 0.84); and an electrocardiogram with sinus rhythm and no abnormalities.

On the third day of hospitalization, the patient continued to have fever spikes; therefore, methylprednisolone was started at 2 mg/kg/day, and the ASA dose was increased to 80 mg/kg/day. Subsequently, a reduction in edema of the hands and feet was observed, which became painless on palpation, along with progressive fading of the erythematous maculopapular lesions. Laboratory tests for blood hematology, ESR, CRP, and liver function tests (LFTs) were requested again (Table 1). There was slight improvement in anemia (Hb 11.2 g/dL), a marked increase in leukocytes bordering on leukocytosis (16.9 × 10^3^/µL), persistence of neutrophilia (11.1 × 10^3^/µL), improvement in lymphopenia (4.9 × 10^3^/µL), decrease in acute-phase reactants, persistent increase in fibrinogen (553 mg/dL), and a pronounced elevation of D-dimer (2988 ng/mL) (Table 1).

On the fourth day of hospitalization, the patient was still experiencing fever spikes, which led to suspicion of resistance to IVIG. For this reason, a consultation with the pediatric rheumatology department was requested. The patient was classified as being at high risk for resistance to IVIG (4 points) according to the Kobayashi Score and low risk for coronary aneurysms (3 points) according to the Harada Score. Consequently, it was decided to continue steroid therapy and administer a second dose of IVIG. Twelve hours after administration, fever spikes, conjunctival injection, and erythema resolved, with a significant reduction in extremity edema.

### Outcome

On the fifth day of hospitalization, the patient was fever-free for 36 h, and her skin lesions decreased. The patient no longer had conjunctival injections, her lips remained swollen and cracked, and the edema had decreased. On the sixth day, she had gone 72 h without a fever, so the ASA dose was reduced to 5 mg/kg/day, and the rash had almost completely disappeared, with edema limited to the lower limbs. The patient was referred to a tertiary care facility where a second echocardiogram was performed by the pediatric cardiology service, which reported a structurally healthy heart, preserved biventricular systolic function, and no coronary abnormalities, although she was still in the subacute stage and was scheduled for a new evaluation in one month. On her eighth day of hospitalization, with no further fever and clinically significant improvement in the rash, mucosal lesions, and edema, laboratory tests were performed again (Table 1), revealing persistent anemia (Hb: 11 g/dL), mild thrombocytosis (platelets: 496 × 10^3^/µL), resolution of neutrophilia (2.8 × 10^3^/µL), recurrence of lymphopenia (1.6 × 10^3^/µL), improvement in CRP (5.39 mg/dL), decreased D-dimer (1507 ng/mL), and increased ferritin (149.06 ng/mL). She was therefore discharged from the hospital with a follow-up appointment at the outpatient clinic for evaluation by the cardiology and pediatrics services.

## 3. Discussion

Atypical cases of KD require considerable clinical expertise for timely diagnosis and treatment because clinical findings can be “subtle and fleeting,” the risk of developing coronary artery disease is very high, and the probable differential diagnoses are diverse. Compared to the classic form, our patient did not meet the five conventional diagnostic criteria, so it was necessary to apply the laboratory criteria proposed by the AHA for atypical forms of this disease [1]. The main criteria for atypical KD include fever for 5 days (or more) and 2 major clinical symptoms: CRP > 3.0 mg/dL or ESR 40 mm/h or, in its absence, at least 3 of the following 6 additional laboratory criteria: anemia for age, platelet count of 450,000/mm^3^ after the 7th day of fever, albumin 3.0 g/dL, elevated alanine transaminase, white blood cell count of 15,000/mm^3^, urine white blood cells ≥10/high-power field [5]. Our patient completed 5 days of fever within the hospital despite medication, bilateral non-purulent conjunctival injection, changes in the oral mucosa, and rash as clinical criteria for KD; laboratory tests showed elevation of acute-phase reactants (CRP: 11.10 mg/dL and ESR: 45 mm/h). Although rhinovirus was detected in our patient, this finding should be interpreted with caution. The assay used (FilmArray^®^) detects rhinovirus/enterovirus without species-level differentiation, and rhinovirus is a common pathogen in children. Therefore, its detection does not establish causality for KD [27]. The possibility of coincidental carriage or unrelated viral infection cannot be excluded [28].

There has been a growing trend toward understanding the pathophysiology of diseases from an immunological perspective. In KD, an essential factor is the activation of multiple innate and adaptive components of the immune system [29]. Immunological assessment through qRT-PCR has revealed a profile marked by the overexpression of proinflammatory cytokines and type I interferons during the acute phase [13,30,31], among which IL-1, IL-2, IL-4, IL-6, IL-8, IL-10, IL-17, IFN-γ, and TNF-α stand out as the main ones involved [13,14,15,18,32,33]. Recent studies, such as those documented, show distinct immunological patterns between classic KD, incomplete forms, MIS-C, and other systemic vasculitis [11,18]. In MIS-C, there is a predominance of elevated levels of IL-6, IL-10, and IL-17A, TNF, IL-1β, IFN-γ, IL-2, or chemokines induced by IFN-γ [14]. Elevated levels of TNF-α, IFN-γ, IL-1β, IL-6, and IL-10 have been associated with more pronounced inflammatory phenotypes and more aggressive disease progression with a higher risk of developing KD shock syndrome (KDSS) [13]. However, other studies report that the cytokine storms in KD and KDSS are similar, and the clinical influence is the overexpression [34]. Hence, the importance of early diagnosis and administration of IVIG during the acute phase (within 10 days of disease onset), at a dose of 2 g/kg in a 10–12 h infusion. This intervention reduces the incidence of coronary artery aneurysms to less than 5% [1,15,35], which was a successful intervention in our patient. However, once the acute febrile phase of KD resolves, the clinical focus shifts from suppressing systemic inflammation to long-term monitoring and management [36]. The primary objectives of outpatient care are to prevent thrombotic complications in the affected coronary arteries, monitor the progression of coronary arterial lesions, and mitigate the long-term risk of premature atherosclerotic cardiovascular disease [37]. Echocardiography is the cornerstone of follow-up, as it allows measurement of coronary Z-scores, detection of aneurysms, assessment of ventricular function, and identification of pericardial effusions [36,37]. Follow-up echocardiograms are recommended at 1–2 weeks and again at 4–6 weeks after treatment to establish the maximum coronary dimensions and assign a final risk level [36,38]. Outpatient pharmacological management is dynamic and risk-adapted. In low-risk patients, therapy is gradually de-escalated [36], whereas in high-risk patients, an intensified and long-term combination regimen is required, reflecting a highly individualized strategy [36]. Aspirin is administered at high (80–100 mg/kg/day) or moderate (30–50 mg/kg/day) doses for its anti-inflammatory effect until the patient has been afebrile for 48–72 h [37]. Once inflammation is controlled, the main residual risk is coronary thrombosis [39], and the aspirin dose is reduced to a low antiplatelet regimen (3–5 mg/kg/day) [36]. Patients with large or giant aneurysms (Level IV) or coronary obstruction (Level V) require more aggressive antithrombotic prophylaxis due to turbulent flow and endothelial dysfunction [36]. This was not the case in our patient. Corticosteroids (e.g., methylprednisolone, prednisolone) are used as adjuvant therapy in the acute phase for high-risk patients or those resistant to initial IVIG treatment [40]. When indicated, they are not given as a single pulse but as a tapering course over 2–3 weeks, often initiated in-hospital and continued after discharge [36,40]. A typical regimen may involve intravenous methylprednisolone followed by oral prednisolone, with progressive dose reduction until discontinuation [41]. Patients discharged on steroid tapering require close outpatient follow-up to monitor adverse effects and ensure safe withdrawal [41]. Studies suggest that the immunomodulatory effects of IVIG derive from the interaction of various components of the immune system targeting cells of the innate immune system (neutrophils, monocytes, macrophages, dendritic cells, natural killer cells, and natural killer T cells) or adaptive immune system (B or T lymphocytes), as well as the vascular endothelial system, with the immune response being activated by PAMPs (pathogen-associated molecular patterns) and DAMPs (damage-associated molecular patterns). PAMPs (derived from specific microbial populations) originate from invariant molecular sequences, whereas DAMPs are derived from cells released in response to tissue damage. Both molecules bind to pentraxins, collectins (mannose-binding lectins), and M-ficolins, which are pattern recognition molecules (PRMs) that can activate complement pathways, inducing an innate immune response [35]. Acute hyperinflammation in KD is mediated by an imbalance between the increased responses of T helper 17 (Th17)/Th1 with high levels of IL-17, associated with high activity in the neutrophils producing the release of reactive oxygen species, causing injury to endothelial cells [14,34]. The neutrophil extracellular traps (NETs) are also implicated in the pathogenesis of KD. NETs play a protective role against infections as components of the innate immune system, but they have a high pathogenic potential for immune dysfunction and for promoting inflammation and tissue damage, a mechanism observed in multiple autoimmune diseases [14]. In addition, some patients with KD present with a phagocytic activation syndrome due to the overproduction of IFN-γ and its downstream mediators, interferon-inducible protein (IP)-10 and TNF-α. This finding, seen in autopsy studies, is associated with early necrotizing vasculitis due to activation of innate phagocytes, followed by coronary remodeling secondary to thrombosis with lymphocyte infiltration and formation of aneurysms in the adaptive immunity phase [34]. It has been described that IL-17 induces key proinflammatory cytokines in this pathology, such as IL-6, IL-8, TNF-α, and IL-1b, perpetuating tissue inflammation [18]. The case presented in this study also reveals prominent activation of the IL-1/IL-8 axis and type I interferons, without elevation of TNF-α, which may reflect secondary downregulation or an alternative phase of immune activation. The absence of eosinophilia in this case, despite high IL-5 expression, suggests an activated Th2 component without peripheral hematological translation, a phenomenon described in some phases of certain viral inflammatory responses. Furthermore, the downregulation of IL-2 and IL-4, together with the activation of IL-12 and IFN-γ, supports a trend toward a dominant Th1 immune response. Our patient presented with persistent fever, which was considered to be IVIG-resistant KD, the prevalence of which varies between 10% and 20% [35], confirmed by the Kobayashi score, for which corticosteroid treatment was indicated. There are individual parameters that have been associated with a higher risk of resistance, such as age under 12 months, hypoalbuminemia, transaminasemia, and neutrophilic leukocytosis, as well as genetic factors such as single nucleotide polymorphisms (SNPs) in IFN-γ, DC-SIGN, IL-1B, MRP4, BAZ1A, STX1B, high mobility group box 1 (HMGB1), and P2Y12 (P2RY12) genes, whose children require more aggressive therapies with monoclonal antibodies and immunomodulators [35]. Significantly higher levels of IL-6 and IL-10 were observed in patients who did not respond to IVIG and in those who developed aneurysms in CA [13,18]. IL-17A has also been implicated in IVIG resistance [18]. It has been reported that a higher ratio of neutrophils to lymphocytes (N/L) implies an imbalance between proinflammatory responses and immune regulation; therefore, the polarization of T cells leans towards the Th2 pathway in response to IVIG therapy. Thus, a high eosinophil count and elevated levels of IL-5 are favorable markers for the success of IVIG treatment [34]. IL-6 has been implicated in myocardial injury in patients with COVID-19, and IL-1 plays an important role in the inflammation of endothelial cells in coronary arteries [15].

Techniques such as qRT-PCR have proven useful for the discovery of biomarkers [42] to speed up early diagnosis and treatment due to their high sensitivity, specificity, and accuracy in their results. However, there are still limitations to their use, such as sample storage problems, patient-specific conditions such as nutritional status, long waiting periods, the need for large equipment, and professional requirements for operators [42,43]. This facilitates the way for the search for standardization of this type of testing, implementation in disease diagnostic criteria, and unification of expression reference values in PBMCs for clinical practice, based on expert consensus and clinical trials. It is essential to know that early detection and treatment of any disease significantly limit the complications that may arise from it. As shown above, in cases such as KD, time plays an important role, especially if it is present in atypical forms, where delayed treatment leads to severe cardiovascular complications. Hence, exploring new diagnostic approaches using molecular techniques such as qRT-PCR is of interest. In this context, our study employed a dual-compartment strategy: cytokine mRNA expression was quantified in PBMCs isolated using Histopaque^®^-1077, while protein concentrations were assessed in serum. Although these compartments are biologically distinct, their combined analysis provides complementary insight. PBMC-derived transcripts offer cell-type-specific resolution, reflecting immune activation in mononuclear cells, whereas circulating cytokines integrate broader systemic contributions from diverse cell types, including epithelial, endothelial, and stromal sources. This is evident in our findings: IL-5 was markedly upregulated at the mRNA level (6.68 log 2 fold change), yet circulating protein levels remained within normal serum reference ranges (8.24 pg/mL). Similarly, TNF-α showed both transcriptional downregulation (−0.22 log 2 fold change) and low–normal circulating protein (0.88 pg/mL). These patterns are not contradictory but rather may reflect physiological compartmentalization and regulatory kinetics. mRNA-based quantification is particularly informative in early or localized immune responses, where systemic protein release may not yet be substantial. Therefore, we argue that qRT-PCR data from PBMCs, while not directly interchangeable with serum protein levels, serve as a sensitive indicator of immunological shifts relevant to diagnosis and pathophysiology in atypical KD. In the same sense, it is important to note that, due to the heterogeneity of the cellular composition within PBMCs, it is not possible to determine which specific subpopulation is responsible for the observed gene expression pattern. The transcriptomic signatures may predominantly reflect the activity of certain immune cell types, such as plasmacytoid dendritic cells or monocytes, but this cannot be confirmed based on our current data [44,45].

Based on the findings obtained in this clinical case, we suggest that a qRT-PCR immunological profile from PBMCs might complement routine laboratory tests in atypical forms of KD. The observed pattern of elevated IFN-α/β, IL-1β, IL-8, IL-12, and IFN-γ, together with IL-2/IL-4 suppression and low TNF-α, should be considered a hypothesis-generating observation that requires validation in prospective cohorts. Importantly, such molecular panels could complement, but not replace, established clinical and laboratory diagnostic criteria.

## 4. Conclusions

This case supports the importance of considering atypical or incomplete KD in pediatric patients presenting with persistent fever and systemic inflammation, even in the absence of the five classic diagnostic criteria. The incorporation of immunological biomarkers via qRT-PCR allowed for the identification of a characteristic proinflammatory profile, with overexpression of type I interferons, IL-1, IL-8, and IFN-γ and downregulation of TNF-α, IL-2, and IL-4. These findings provide additional evidence to support diagnosis in a clinical context limited by the absence of cardinal clinical signs. The use of a cytokine panel via qRT-PCR represents a key differentiator from previously reported cases in the literature, where this type of analysis is rarely incorporated into the evaluation of atypical KD. This approach enables characterization of a mixed immunological phenotype characterized by Th1 and interferon dominance, which could serve as a complementary tool for early therapeutic decision-making. We emphasize the need to develop integrative diagnostic strategies that include immunological profiles in patients with clinical suspicion of pediatric vasculitis lacking traditional diagnostic criteria in order to reduce cardiovascular risk and improve prognosis. Although mRNA and protein levels may diverge due to distinct cellular sources and regulatory mechanisms, the combined analysis of cytokine transcripts in PBMCs and serum protein concentrations provides complementary insight. In this case, transcriptional profiling revealed early immune activation not fully captured by systemic measurements, reinforcing the utility of molecular profiling in the assessment of atypical KD. However, this report reflects a single-case observation and is limited by the evaluation at a single time point; therefore, prospective validation in larger cohorts is warranted. qRT-PCR cytokine panels should be considered as complementary tools to existing clinical and laboratory criteria but not as replacements.

## Figures and Tables

**Table 1:** Chronology of biochemical laboratory variables.

Biochemical Variable	Reference Values	Results		
		Admission	Day 3 (HS)	Day 8 (HD)
Erythrocytes (×10^6^ g/dL)	3.9–4.6	4.18	4.30	4.19
Hemoglobin (gr/dL)	11.5–12.5	10.9 *	11.2 *	11 *
Hematocrit (%)	34–37	32.70 *	33.4 *	32.2 *
Mean corpuscular volume (fL)	75–81	78.3	77.6	76.9
Mean corpuscular hemoglobin (pg)	25–31	26.1	26	26.3
RDW-CV	12–14	14.3 *	14.2 *	14.7 *
Platelets (×10^3^/µL)	150–350	278	332	495 *
Leukocytes (K/µL)	6–17	11.8	16.9	4.5
Neutrophils (×10^3^/µL)	1.5–8.5	9.2 *	11.1 *	2.8
Lymphocytes (×10^3^/mm^3^)	3–9.5	1.7 *	4.9	1.6 *
Monocytes (×10^3^/mm^3^)	0.5	0.6	0.9	0.1
Eosinophils (×10^3^/mm^3^)	0.3	0.2	0.1	0.0
Basophiles (×10^3^/µL)	0.001–0.005	0.0	0.0	0.0
C-reactive protein (mg/dL)	0–0.5	11.10 *	9.59 *	5.39 *
Erythrocyte sedimentation rate (mm/h)	4–20	45 *	24 *	NT
Prothrombin time (seconds)	12.1–14.5	13.1	NT	NT
Partial thromboplastin time (s)	33.6–43.8	66.2 *	NT	NT
Fibrinogen (mg/dL)	162–401	468 *	553 *	376
D-dimer (ng/mL)	90–530	484	2988 *	1507 *
Glucose (mg/dL)	60–100	101 *	NT	NT
Urea (mg/dL)	15–38	30.6	NT	NT
Urea nitrogen (mg/dL)	5–18	14.3	NT	NT
Creatinine (mg/dL)	0.3–0.7	0.35	NT	NT
Direct bilirubin (mg/dL)	0 < 0.2	0.139	0.122	0.10
Indirect bilirubin (mg/dL)	0–1	0.21 *	0.18	0.04
Total bilirubin (mg/dL)	0.3–1.2	0.35	0.31	0.14
Albumin (g/dL)	3.6–5.2	4.6	4.1	3.8
Total protein (g/dL)	5.6–7.5	6.9	6.5	8.2
Globulins (g/dL)	2–3	2.3	2.4	4.4
Alkaline phosphatase (U/L)	100–320	343.5 *	212.9	194.4
Alanine aminotransferase (U/L)	5–30	35 *	8.7	9.4
Lactic dehydrogenase (U/L)	110–295	290	294	238
Aspartate aminotransferase (U/L)	13–35	35	23	29
Creatine kinase (U/L)	20–180	82.5	NT	29.2
Creatine kinase MB fraction (U/L)	23–95	22.79	NT	27.58
Sodium (mEq/L)	135–147	137	NT	NT
Chlorine (mEq/L)	97–107	106.36	NT	NT
Potassium (mEq/L)	3.4–4.7	4.37	NT	NT
Magnesium (mg/dL)	1.6–2.4	2.02	NT	NT
Phosphorus (mg/dL)	4–6.5	3.6	NT	NT
Serum calcium (mg/dL)	8.8–10.8	9.29	NT	NT
Ferritin (ng/mL)	7–140	74.98	NT	149.06 *
c-ANCA (U/L)	Negative: <20 Positive: >20	12.5	NT	NT
p-ANCA (U/L)	Negative: <20 Positive: >20	<3.20	NT	NT
E-Selectin (ng/mL)	18.5–35	33.5	NT	NT
Interleukin 6 (pg/mL)	0.0–7.0	2.19	NT	NT
Interleukin 5 (pg/mL)	2.0–10.0	8.24	NT	NT
Immunoglobulin E (U/mL)	0.31–29.5	39.4 *	NT	NT
TNF-α (pg/mL)	0.5–1.40	0.88	NT	NT
Myoglobin (ng/mL)	11.8–30.43	14.1	NT	17.2
Troponin I (ng/mL)	0.004–0.021	0.03	NT	NT

Reference data were obtained from [19,20,21,22,23,24,25]. Values outside the reference range are highlighted with an asterisk. c-ANCA: Cytoplasmic anti-neutrophil cytoplasmic antibodies; p-ANCA: Perinuclear anti-neutrophil cytoplasmic antibodies; NT: Not repeated; TNF-α: Tumoral necrosis factor-alfa; RDW-CV: Red Cell Distribution Width Coefficient of Variation; HS: Hospital stay; HD: Hospital discharge. * Values outside the reference range.

**Table 2:** General urine test of the patient with atypical Kawasaki disease.

Urine Test	Result	Reference Value
Physical-chemical test		
Color	Yellow	Amber/Yellow
Appearance	Slightly cloudy	Translucent
pH	6	5–6.5
Density	1.015	1.002–1.030
Erythrocytes	Negative	Negative
Leukocytes	Negative	Negative
Nitrites	Negative	Negative
Proteins	Negative	Negative
Bilirubin	Negative	Negative
Ketones	20 mg/dL *	<8 mg/dL
Glucose	Negative	Negative
Urobilinogen	Negative	1 mg/dL
Microscopic examination		
Leukocytes (per field)	3–5	0–5
Erythrocytes (per field)	0–1	0–2
Bacteria (per field)	Scarce	Negative
Mucoid filament	Scarce	Negative
Yeasts (per field)	Negative	Negative
Cylinders (per field)	Negative	0–1
Crystals (per field)	Negative	Negative

Reference data were obtained from [20,26]. Values outside the reference range are highlighted with an asterisk. Sample obtained on the first day of hospitalization.

**Table 3:** Comparative cytokine expression profiles in pediatric vasculitis.

Gene	Expression Level (This Study)	Kawasaki Disease	Cutaneous Leukocytoclastic Vasculitis	Eosinophilic Vasculitis (Churg–Strauss)	Childhood-onset SLE	Viral Infection with Post-Immune Phenomenon
IL-2	−1.69	↓/↔	↓	↓	↓	↓
IL-4	−1.41	↔	↔	↑	↑	↓
TNF-α	−0.22	↑	↑	↑	↑	↔/↑
NF-κB	0.21	↑	↑	↑	↑	↑
IL-6	0.77	↑↑	↑	↑	↑↑	↑
IL-12	1.03	↑	↑	↔/↑	↔	↑
IL-8	1.64	↑↑	↑↑	↑	↑	↑
IFN-γ	2.94	↑	↑	↑	↑	↑↑
IL-1β	2.96	↑	↑↑	↑	↑	↑
IFN-β	6.3	↔	↔	↔	↑↑	↑↑
IL-5	6.68	↔	↔	↑↑↑	↑	↑
IL-1α	7.19	↑	↑↑	↑	↑	↑
IFN-α	7.71	↔	↔	↔	↑↑↑	↑↑

Upward arrows (↑) indicate upregulation, downward arrows (↓) indicate downregulation, and horizontal arrows (↔) denote baseline (unchanged) expression levels. Patient qRT-PCR values are presented in the first column for direct comparison. IL: Interleukin; TNF-α: Tumor necrosis factor-alpha; IFN-α/β/γ: Interferon alpha/beta/gamma; NF-κB: Nuclear factor kappa-light-chain enhancer of activated B cells; KD: Kawasaki disease; MIS-C: Multisystem inflammatory syndrome in children.

**Table 4:** Viral panel from a nasopharyngeal sample of a patient with atypical Kawasaki disease.

Pathogen	Day Result	Result
Human parainfluenza virus type 2 (HPIV-2)	Day 4	Negative
Human parainfluenza virus type 3 (HPIV-3)	Day 4	Negative
Human parainfluenza virus type 4 (HPIV-4)	Day 4	Negative
Human adenovirus (HAdV)	Day 4	Negative
Human coronavirus 229E (HCoV-229E)	Day 4	Negative
Human rhinovirus (HRV)	Day 4	Positive

## Data Availability

The original contributions presented in this study are included in the article. Further inquiries can be directed to the corresponding authors.

## Abbreviations

The following abbreviations are used in this manuscript:

AHA	American Heart Association
ASA	acetylsalicylic acid
BH	hematological biometrics
CA	coronary arteries
c-ANCA	Cytoplasmic anti-neutrophil cytoplasmic antibodies
cDNA	Complementary DNA
CRP	C-Reactive Protein
DAMPs	damage-associated molecular patterns
ESR	erythrocyte sedimentation rate
Hb	Hemoglobin
HD	Hospital discharge
HS	Hospital stays
IFN-α or INF-a	Interferon alpha
IFN-β or INF-b	Interferon beta
IFN-γ or INF-g	Interferon gamma
IGIV	Intravenous immunoglobulin
IL	Interleukin
IL-1α	Interleukin 1-alpha
IL-1β	Interleukin 1 beta
KD	Kawasaki disease
KDSS	Kawasaki disease shock syndrome
miRNA	MicroRNA
MIS-C	Multisystem inflammatory syndrome in children
NF-κB	Factor nuclear kappa B
NETs	Neutrophil extracellular trap
p-ANCA	Perinuclear anti-neutrophil cytoplasmic antibodies
PAMPs	pathogen-associated molecular pattern
Plt	Platelets
qRT-PCR	Quantitative Reverse Transcription Polymerase Chain Reaction
RDW-CV	Red Cell Distribution Width Coefficient of Variation
TNF-α	Tumor necrosis factor-alpha

## References

[1] Jone P.N., Tremoulet A., Choueiter N., Dominguez S.R., Harahsheh A.S., Mitani Y., Zimmerman M., Lin M.T., Friedman K.G., American Heart Association Rheumatic Fever E. (2024). Update on Diagnosis and Management of Kawasaki Disease: A Scientific Statement From the American Heart Association. Circulation.

[2] Singh S., Vignesh P., Burgner D. (2015). The epidemiology of Kawasaki disease: A global update. Arch. Dis. Child..

[3] Newburger J.W., Takahashi M., Burns J.C. (2016). Kawasaki Disease. J. Am. Coll. Cardiol..

[4] Kainth R., Shah P. (2021). Kawasaki disease: Origins and evolution. Arch. Dis. Child..

[5] Kuo H.C. (2023). Diagnosis, Progress, and Treatment Update of Kawasaki Disease. Int. J. Mol. Sci..

[6] Maggio M.C., Corsello G. (2015). Atypical and incomplete Kawasaki disease. Ital. J. Pediatr..

[7] Yu J.J. (2012). Diagnosis of incomplete Kawasaki disease. Korean J. Pediatr..

[8] Sonobe T., Kiyosawa N., Tsuchiya K., Aso S., Imada Y., Imai Y., Yashiro M., Nakamura Y., Yanagawa H. (2007). Prevalence of coronary artery abnormality in incomplete Kawasaki disease. Pediatr. Int..

[9] Dozois C.M., Oswald E., Gautier N., Serthelon J.P., Fairbrother J.M., Oswald I.P. (1997). A reverse transcription-polymerase chain reaction method to analyze porcine cytokine gene expression. Vet. Immunol. Immunopathol..

[10] Berti R., Williams A.J., Moffett J.R., Hale S.L., Velarde L.C., Elliott P.J., Yao C., Dave J.R., Tortella F.C. (2002). Quantitative real-time RT-PCR analysis of inflammatory gene expression associated with ischemia-reperfusion brain injury. J. Cereb. Blood Flow. Metab..

[11] Cannon L., Campbell M.J., Wu E.Y. (2023). Multisystemic Inflammatory Syndrome in Children and Kawasaki Disease: Parallels in Pathogenesis and Treatment. Curr. Allergy Asthma Rep..

[12] Kuiper R., Wright V.J., Habgood-Coote D., Shimizu C., Huigh D., Tremoulet A.H., van Keulen D., Hoggart C.J., Rodriguez-Manzano J., Herberg J.A. (2023). Bridging a diagnostic Kawasaki disease classifier from a microarray platform to a qRT-PCR assay. Pediatr. Res..

[13] Li Y., Zheng Q., Zou L., Wu J., Guo L., Teng L., Zheng R., Jung L.K.L., Lu M. (2019). Kawasaki disease shock syndrome: Clinical characteristics and possible use of IL-6, IL-10 and IFN-gamma as biomarkers for early recognition. Pediatr. Rheumatol. Online J..

[14] Sharma C., Ganigara M., Galeotti C., Burns J., Berganza F.M., Hayes D.A., Singh-Grewal D., Bharath S., Sajjan S., Bayry J. (2021). Multisystem inflammatory syndrome in children and Kawasaki disease: A critical comparison. Nat. Rev. Rheumatol..

[15] Bordea M.A., Costache C., Grama A., Florian A.I., Lupan I., Samasca G., Deleanu D., Makovicky P., Makovicky P., Rimarova K. (2022). Cytokine cascade in Kawasaki disease versus Kawasaki-like syndrome. Physiol. Res..

[16] Lee S.B., Kim Y.H., Hyun M.C., Kim Y.H., Kim H.S., Lee Y.H. (2015). T-Helper Cytokine Profiles in Patients with Kawasaki Disease. Korean Circ. J..

[17] Eberhard B.A., Andersson U., Laxer R.M., Rose V., Silverman E.D. (1995). Evaluation of the cytokine response in Kawasaki disease. Pediatr. Infect. Dis. J..

[18] Jia S., Li C., Wang G., Yang J., Zu Y. (2010). The T helper type 17/regulatory T cell imbalance in patients with acute Kawasaki disease. Clin. Exp. Immunol..

[19] Jorge H.A. (2019). Hematología Práctica: Interpretación del Hemograma y de las Pruebas de Coagulación, AEPap, Ed..

[20] Vargas Y.G. (2014). Valores de Referencia en Pediatría.

[21] Merino A.H. (2012). Anemias en la infancia y adolescencia. Clasificación y diagnóstico. Pediatr. Integral.

[22] Dossybayeva K., Bexeitov Y., Mukusheva Z., Almukhamedova Z., Assylbekova M., Abdukhakimova D., Rakhimzhanova M., Poddighe D. (2022). Analysis of Peripheral Blood Basophils in Pediatric Systemic Lupus Erythematosus. Diagnostics.

[23] Anderson C.C., Sunaina K., Mark Tiffany E. (2024). The Harriet Lane Handbook: A Manual for Pediatric House Officers.

[24] Doven S.S., Tezol O., Yesil E., Durak F., Misirlioglu M., Alakaya M., Karahan F., Killi I., Akca M., Erdogan S. (2024). The 2023 Turkiye-Syria earthquakes: Analysis of pediatric victims with crush syndrome and acute kidney Injury. Pediatr. Nephrol..

[25] McEvoy J.W., Wang D., Brady T., Tang O., Ndumele C.E., Coresh J., Christenson R.H., Selvin E. (2023). Myocardial Injury Thresholds for 4 High-Sensitivity Troponin Assays in a Population-Based Sample of US Children and Adolescents. Circulation.

[26] Cone T.E. (1968). Diagnosis and treatment: Some syndromes, diseases, and conditions associated with abnormal coloration of the urine or diaper: A Clinician’s Viewpoint. Pediatrics.

[27] Marutani K., Murata K., Mizuno Y., Onoyama S., Hoshina T., Yamamura K., Furuno K., Sakai Y., Kishimoto J., Kusuhura K. (2024). Respiratory viral infections and Kawasaki disease: A molecular epidemiological analysis. J. Microbiol. Immunol. Infect..

[28] Turnier J.L., Anderson M.S., Heizer H.R., Jone P.-N., Glodé M.P., Dominguez S.R. (2015). Concurrent respiratory viruses and Kawasaki disease. Pediatrics.

[29] Menikou S., Langford P.R., Levin M. (2019). Kawasaki disease: The role of immune complexes revisited. Front. Immunol..

[30] Huang J., Wu S., Cao S., Zhu X., Zhang S. (2020). Neutrophil-Derived Semaphorin 4D Induces Inflammatory Cytokine Production of Endothelial Cells via Different Plexin Receptors in Kawasaki Disease. Biomed. Res. Int..

[31] Macchia I., La Sorsa V., Urbani F., Moretti S., Antonucci C., Afferni C., Schiavoni G. (2023). Eosinophils as potential biomarkers in respiratory viral infections. Front. Immunol..

[32] Lee K.H., Li H., Lee M.H., Park S.J., Kim J.S., Han Y.J., Cho K., Ha B., Kim S.J., Jacob L. (2022). Clinical characteristics and treatments of multi-system inflammatory syndrome in children: A systematic review. Eur. Rev. Med. Pharmacol. Sci..

[33] Huang H.-C., Kuo H.-C., Yu H.-R., Huang H.-C., Chang J.-C., Lin I.-C., Chen I.-L. (2021). Profile of urinary cytokines in Kawasaki disease: Non-invasive markers. Diagnostics.

[34] Chang L., Yang H.-W., Lin T.-Y., Yang K.D. (2021). Perspective of immunopathogenesis and immunotherapies for Kawasaki disease. Front. Pediatr..

[35] Nadig P.L., Joshi V., Pilania R.K., Kumrah R., Kabeerdoss J., Sharma S., Suri D., Rawat A., Singh S. (2023). Intravenous immunoglobulin in Kawasaki Disease—Evolution and pathogenic mechanisms. Diagnostics.

[36] McCrindle B.W., Rowley A.H., Newburger J.W., Burns J.C., Bolger A.F., Gewitz M., Baker A.L., Jackson M.A., Takahashi M., Shah P.B. (2017). Diagnosis, treatment, and long-term management of Kawasaki disease: A scientific statement for health professionals from the American Heart Association. Circulation.

[37] Saguil A., Fargo M., Grogan S. (2015). Diagnosis and management of Kawasaki disease. Am. Fam. Physician.

[38] Siraj S., Parra C.S., Jordan J., Shah A., Amankwah E., Fadrowski J. (2021). Clinical Utility of Follow-up Echocardiograms in Uncomplicated Kawasaki Disease. Sci. J. Clin. Med..

[39] Kim S.H. (2021). Diagnosis of coronary artery abnormalities in Kawasaki disease: Recent guidelines and z score systems. Clin. Exp. Pediatr..

[40] de Graeff N., Groot N., Ozen S., Eleftheriou D., Avcin T., Bader-Meunier B., Dolezalova P., Feldman B.M., Kone-Paut I., Lahdenne P. (2019). European consensus-based recommendations for the diagnosis and treatment of Kawasaki disease–the SHARE initiative. Rheumatology.

[41] Crespo-Sánchez V., Anda-Gómez M., García-Campos J., Lazcano-Bautista S., Mendiola-Ramírez K., Valenzuela-Flores A. (2015). Diagnóstico y Tratamiento de la Enfermedad de Kawasaki en el Primero, Segundo y Tercer Nivel de Atención PL México, DF.

[42] Ban E., Song E.J. (2022). Considerations and suggestions for the reliable analysis of miRNA in plasma using qRT-PCR. Genes.

[43] Wang Y., Chen J., Yang Z., Wang X., Zhang Y., Chen M., Ming Z., Zhang K., Zhang D., Zheng L. (2024). Advances in nucleic acid assays for infectious disease: The role of microfluidic technology. Molecules.

[44] Bolen C.R., Uduman M., Kleinstein S.H. (2011). Cell subset prediction for blood genomic studies. BMC Bioinform..

[45] Zhang J., Zhuang W., Li Y., Deng C., Xuan J., Sun Y., He Y. (2025). Bioinformatic analysis and experimental verification reveal expansion of monocyte subsets with an interferon signature in systemic lupus erythematosus patients. Arthritis Res. Ther..

